# A static precise single-point positioning method based on carrier phase zero-baseline self-differencing

**DOI:** 10.1038/s41598-024-63570-2

**Published:** 2024-06-01

**Authors:** Kaihui Lv, Chenglin Cai, Yihao Cai, Wenhui Guan, Zexian Li, Mingjie Wu, Lingfeng Cheng

**Affiliations:** 1 School of Mathematics and Computational Science, Xiangtan University, Xiangtan, 411105 Hunan China; 2 School of Automation and Electronic Information, Xiangtan University, Xiangtan, 411105 Hunan China; 3 School of Materials Science and Engineering, Xiangtan University, Xiangtan, 411105 Hunan China; 4 Shanghai Astronomical Observatory, Chinese Academy of Sciences, Shanghai, 200030 China; 5 School of Physical Science and Technology, ShanghaiTech University, Shanghai, 200030 China

**Keywords:** Precise point positioning, Rapid convergence, Doppler principle, Zero-baseline self-differencing, Applied mathematics, Computational astrophysics, Aerospace engineering

## Abstract

Satellite navigation positioning has become an indispensable component of everyday life, where precise pinpointing and rapid convergence are crucial in delivering timely and accurate location information. However, due to the damping of integer ambiguities and system residual errors, the rapid convergence of Precise Point Positioning (PPP) implementation is a significant challenge. To address this, this paper proposes a novel Carrier Phase Zero-Baseline Self-Differencing Precise Point Positioning (CZS-PPP) technique and its ionosphere-free fusion model. By employing the proposed CZS-PPP approach in separate scenarios involving BDS-3, GPS, and dual-system settings, we systematically validate the efficacy of the method. The experimental results indicate that the convergence time of the method is less than 4 min in a single-system scenario. Furthermore, in a dual-system scenario, the method can achieve rapid convergence in less than 3 min. The CZS-PPP technique presented demonstrates the elimination of integer ambiguities and the effective suppression of system residuals, in comparison to the conventional method. The proposed approach has demonstrated remarkable performance across different systems, offering a promising new pathway for achieving PPP fast convergence in BDS/GNSS.

## Introduction

For over 30 years, the development of PPP has progressed slowly. The lengthy convergence time of PPP has posed an international challenge, impeding its adoption in the scientific, academic, and industrial communities and preventing its real-time and commercial use. To address the issue of shortening PPP convergence time effectively, international scholars have proposed several classic PPP models, including the Single-Difference (SD) model^1,2^, the Uncombined (UC) positioning model^3^, the University of Calgary model (UoFC)^4^, and the ionosphere-free combination model (IF)^5–8^. In recent years, with the development of BeiDou Navigation Satellite System (BDS) and Galileo, research on PPP using multi-frequency, multi-system, and multi-frequency multi-system combinations has become a hot topic.

BDS-3 was the first to launch onboard real-time PPP services internationally. Researchers such as Zhang et al. evaluated the static and dynamic positioning performance of PPP-B2b^9^. For BDS-3 (BDS-3 + GPS), the static positioning accuracy was 2.6 (2.1) cm, the dynamic positioning accuracy was 21.5 (15.2) cm horizontally and 33.4 (30.3) cm vertically, with a convergence time of 17.4 (16.2) minutes. Other scholars have reported similar performance results^10–13^. Multi-frequency combination models are advantageous for speeding up PPP convergence^14^. Duong V analyzed the UD model, showing that its convergence time could reach 15 min with positioning accuracy better than 10 cm^15^. Laurichesse D analyzed the positioning performance of three-frequency PPP, demonstrating centimeter-level accuracy^16^. Academician Yang Yuanxi, Basile F, Zhang Xiaohong, and others analyzed the convergence performance of three-frequency PPP. The experimental results indicate that the convergence time of three-frequency PPP can be shortened to 10 minutes^17–19^.

Multi-system combination models also play a significant role in accelerating PPP convergence^20^. The primary benefit of employing multi-system combination lies in the increased number of observed satellites and improved spatial geometry, resulting in enhanced PPP convergence time and positioning accuracy. Noteworthy researchers such as Geng Jianghui, Li Xingxing, and Duong V. have delved into the study of multi-system PPP models, demonstrating that, in comparison to single-system models, multi-system PPP models have the capability to expedite the resolution of integer ambiguities^21,22^. In another exploration, Zhang Baocheng, Li Xingxing, and their colleagues investigated the GCRE four-system PPP model and determined that the static convergence time for the four-system PPP model could be reduced to less than 10 min, with positioning accuracy better than 10 cm^23^. Furthermore, the multi-frequency multi-system combination model has also attracted great attention. The results show that the combination of multi-frequency and multi-system can further shorten the convergence time^24^. In conclusion, diverse combination models can significantly reduce PPP convergence time, albeit facing constraints such as errors in atmospheric delay models, time-varying effects, and gradual changes in GNSS satellite constellations.

In recent years, two new PPP mechanisms, Precise Point Positioning and Real-Time Kinematic (PPP–RTK) and Low Earth Orbit Satellite Navigation Enhanced GNSS (LE-GNSS), have gained significant attention. PPP–RTK aims to overcome the factors impeding rapid convergence in PPP, with a primary focus on effectively resolving errors in atmospheric delay models and their temporal variations^25^. Introduced by Germany’s Wübbena in 2005, this concept relies on ground-based CORS networks to accurately differentiate satellite orbits, satellite clock errors, and atmospheric delay errors. Users can leverage this model data to achieve instantaneous centimeter-level positioning, akin to Real-Time Kinematic (RTK)^26–28^. The “Heavenly RTK” proposed by Hexagon in the United States, essentially disseminates this data via satellites, rendering it a satellite-based PPP–RTK technology. LE-GNSS addresses the slow-changing GNSS satellite constellation structure. Low Earth Orbit (LEO) satellites operate at high speeds, and when combined with BDS-3, they can rapidly alter the geometric structure of GNSS navigation constellations. This approach can solve the ill-posed problem of multi-epoch combined PPP models in a short time frame. Researchers like Li Bofeng and Li Xingxing have conducted studies and analysis, suggesting preliminary conclusions that, with a sufficient number of deployed LEO satellites (300 or more), LE-GNSS can achieve PPP convergence times of less than 1 minute^29,30^.

Harnessing external aids, such as Heavenly RTK and LEO Satellite Navigation Enhancement, undeniably contributes to overcoming the international challenge of PPP slow convergence. This paper seeks to investigate whether PPP can achieve swift convergence autonomously, without relying on external assistance.

## Principles and methods

### CZS-PPP principle

The core principle entails employing itself as a reference, essentially considering the epochs before and after as the reference station and the mobile station. This involves a static baseline length of zero and a dynamic baseline length approaching zero, forming a zero-baseline self-differencing mode. Changes in position relative to the previous epoch are computed by analyzing the carrier phase variations between the current and preceding epochs. This involves subtracting the carrier phase from the previous epoch from the carrier phase of the current epoch. Subsequently, the self-differencing values for carrier phase at each frequency are calculated. The coordinates are then determined using a dual-frequency ionosphere-free combination model.

The basic procedure of our method is as follows: In the first step, Single-Point Positioning (SPP) is used to determine the receiver coordinates at the initial epoch. In the second step, taking the receiver coordinates at the initial epoch as a virtual station, epoch differencing is performed between the second epoch and the initial epoch. The solution provides the coordinate changes relative to the virtual station, so the receiver coordinates at the second epoch are obtained by adding the virtual station coordinates and the coordinate changes. In the third step, the virtual station is updated, taking the receiver coordinates at the second epoch as the new virtual station. Similarly, epoch differencing is performed between the third epoch and the second epoch to obtain new coordinate changes and the receiver coordinates at the third epoch, updating the virtual station. This process is iteratively applied to subsequent epochs until convergence to the true receiver coordinates is achieved. The carrier phase observation equation (converted to distance), as shown in the equation, is used.

1 $$L_{i} = \rho + c \cdot \delta t_{k} - c \cdot \delta t^{j} + G_{h} \cdot T_{h} + G_{w} \cdot T_{w} - I_{i} + N_{i} + \varepsilon_{{L_{i} }}$$

In the above equation, $L_{i}$ represents the carrier phase observations (converted to distance), where the subscript $i$ denotes the carrier frequency. $\rho$ is the satellite-to-ground geometric distance, $\delta t_{k}$ represents the receiver clock bias, $\delta t^{j}$ represents the satellite clock bias, and c is the speed of light, all in units of seconds. $G_{h}$ and $G_{w}$ are the tropospheric zenith direction dry and wet mapping functions, respectively. $T_{h}$ represents the zenith direction dry delay component, obtained from the Saastamoinen model^31^. $T_{w}$ represents the zenith direction wet delay component and is treated as an estimated parameter alongside position parameters. $I_{i}$ represents the ionospheric delay on $L_{i}$. $\varepsilon_{{L_{i} }}$ represents the observation noise for carrier phase, all in units of meters. $N_{i}$ represents the integer ambiguity on $L_{i}$.

The Eq. (1) is differenced between consecutive epochs to obtain the zero-baseline self-differencing equation (converted to distance) as follows:

2 $$\Delta L_{i} = L_{t} - L_{t - 1} = \Delta \rho + c \cdot \left( {\Delta \delta t_{k} - \Delta \delta t^{j} } \right) + G_{h} \cdot \Delta T_{h} + G_{w} \cdot \Delta T_{w} - \Delta I_{i} + \Delta \varepsilon_{{L_{i} }}$$

In the equation, ${\Delta }$ represents the differencing operator between consecutive epochs. The carrier phase zero-baseline self-differencing eliminates the integer ambiguity parameters. ${\Delta }\rho$ represents the change in satellite-to-receiver geometric distance between consecutive epochs, and ${\Delta }\rho$ is defined as follows:

3 $$\Delta \rho = \rho_{t} - \rho_{t - 1} = e_{t} \cdot \left( {R_{t}^{s} - R_{t}^{r} } \right) - e_{t - 1} \cdot \left( {R_{t - 1}^{s} - R_{t - 1}^{r} } \right)$$

In the equation, $t$ and $t - 1$ represent two consecutive epochs; $e_{t} = \frac{{R_{t}^{s} - R_{t}^{r} }}{{\left| {R_{t}^{s} - R_{t}^{r} } \right|}}$ represents the unit direction vector from the receiver to the satellite; $R_{t}^{s}$ represents the satellite coordinate vector, and $R_{t}^{r}$ represents the receiver coordinate vector. Additionally, $R_{t}^{r} = R_{t - 1}^{r} + \Delta R$, where ${ }\Delta R$ represents the change in receiver coordinates. Therefore, ${\Delta }\rho$ can be expressed as follows:

4 $$\Delta \rho = - e_{t} \cdot \Delta R + e_{t} \cdot \left( {R_{t}^{s} - R_{t - 1}^{r} } \right) - e_{t - 1} \cdot \left( {R_{t - 1}^{s} - R_{t - 1}^{r} } \right)$$

Substituting Eq. (4) into Eq. (2) yields:

5 $$\begin{aligned} \Delta L_{i} = & - e_{t} \cdot \Delta R + e_{t} \cdot \left( {R_{t}^{s} - R_{t - 1}^{r} } \right) - e_{t - 1} \cdot \left( {R_{t - 1}^{s} - R_{t - 1}^{r} } \right) + c \cdot \left( {\Delta \delta t_{k} - \Delta \delta t^{j} } \right) \\ & \; + G_{h} \cdot \Delta T_{h} + G_{w} \cdot \Delta T_{w} - \Delta I_{i} + \Delta \varepsilon_{{L_{i} }} \\ \end{aligned}$$

and calculate its error equation as:

6 $$v = \left[ { - e_{x} - e_{y} - e_{z} 1 G_{w} } \right] \cdot \left[ {\begin{array}{*{20}c} {\Delta x} \\ {\Delta y} \\ {\Delta z} \\ {c \cdot \Delta \delta t_{k} } \\ {\Delta T_{w} } \\ \end{array} } \right] - l$$

where $$l$$ is:

7 $$e_{t} = \left( {e_{x} ,e_{y} ,e_{z} } \right) = \left( {\frac{{X^{J} - X_{K}^{0} }}{{\left| {R_{t}^{s} - R_{t}^{r} } \right|}},\frac{{Y^{J} - Y_{K}^{0} }}{{\left| {R_{t}^{s} - R_{t}^{r} } \right|}},\frac{{Z^{J} - Z_{K}^{0} }}{{\left| {R_{t}^{s} - R_{t}^{r} } \right|}}} \right)$$

$\left( {X^{J} ,Y^{J} ,Z^{J} } \right)$ represents the satellite coordinates, while $\left( {X_{K}^{0} ,Y_{K}^{0} ,Z_{K}^{0} } \right)$ represents the approximate coordinates of the receiver.

8 $$\Delta R = \left( {\Delta x,\Delta y,\Delta z} \right)$$

9 $$l = \Delta L_{i} - e_{t} \cdot \left( {R_{t}^{s} - R_{t - 1}^{r} } \right) + e_{t - 1} \cdot \left( {R_{t - 1}^{s} - R_{t - 1}^{r} } \right) + c \cdot \Delta \delta t^{j} - G_{h} \cdot \Delta T_{h} + \Delta I_{i}$$

In Eqs. (6), $v$ represents the residual vector, $l$ denotes the known parameters, and the parameters to be estimated include the changes in receiver coordinates $\Delta R$ for both before and after epochs, the variation in tropospheric zenith wet delay component $\Delta T_{w}$, and the receiver clock bias change $\Delta \delta t_{k}$. When a sufficient number of visible satellites is available, these parameters can be estimated through the least-squares method or Kalman filtering.

### Reconstructed Doppler method

Presently, the unprocessed Doppler observations obtained from receivers display substantial noise, making them unsuitable for accurate GNSS positioning or velocity estimation due to elevated error levels and diminished accuracy. As a result, a method has been suggested to reconstruct Doppler frequency shift values, aiming to produce noise-free theoretical values. The reconstructed alterations in carrier phase demonstrate remarkable precision and are devoid of cycle slips.

The reconstructed Doppler frequency shift values depend on various parameters, such as satellite positions, satellite clock biases, tropospheric delays, ionospheric delays, receiver velocity, receiver position, and receiver clock biases, among others. These parameters constitute the variables to be estimated, establishing a theoretical basis for the pseudorange single-point positioning model utilizing reconstructed Doppler values.

**Figure 1:**
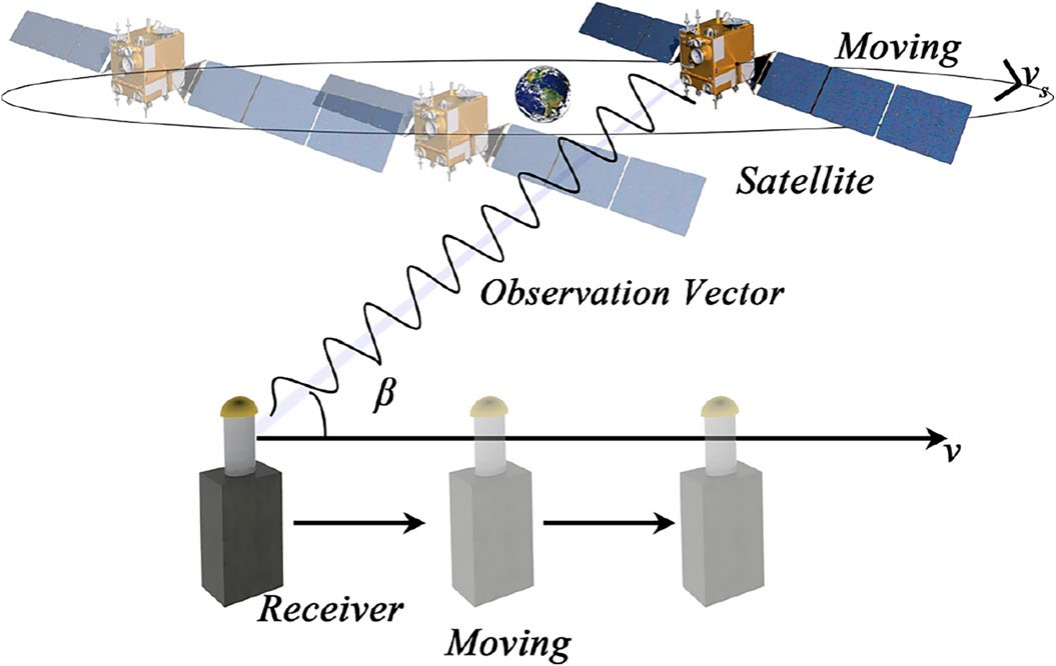
Doppler model of satellites and receivers.

For satellite positioning, satellites orbit around the Earth, making them mobile signal sources. When extending the formula to single-point positioning, one can derive the reconstructed Doppler frequency shift value, denoted as ${ }f_{d}$. In Fig. 1, the observation vector $\overrightarrow {{{ }{\varvec{\rho}}{ }}}$ of a satellite points from the receiver to the satellite, while the $\overrightarrow {{{ }{\varvec{v}}{ }}}$ vector represents the receiver’s velocity. Let $\overrightarrow {{v_{s} }} = (x_{vs} ,{ }y_{vs} ,{ }z_{vs} )$ be the satellite velocity, $P_{s} = (x_{s} ,{ }y_{s} ,{ }z_{s} )$ be the satellite position, and $P_{u} = (x_{u} ,{ }y_{u} ,{ }z_{u} )$ be the receiver’s position, obtained through pseudorange positioning. Therefore, $\vec{\user2{\rho }} = (x_{\rho } ,{ }y_{\rho } ,{ }z_{\rho } ) = \left( {x_{s} - x_{u} ,{ }y_{s} - y_{u} ,{ }z_{s} - z_{u} } \right)$. The reconstructed Doppler value can be calculated as follows:

10 $$f_{d} = \frac{{\left( {\overrightarrow {v} - \overrightarrow {{v_{s} }} } \right) \cdot \overrightarrow {l} }}{\lambda }$$

In the equation, $\vec{\user2{l}}$ is the unit observation vector, $\vec{\user2{l}} = \vec{\user2{\rho }}/\left| {\vec{\user2{\rho }}} \right|$, where $\left| {\vec{\user2{\rho }}} \right|$ represents the satellite-to-earth geometric distance, $\lambda$ is the wavelength. The dot symbol “.”denotes vector dot product.

Due to the presence of cycle slips, clock jumps, and gross errors in traditional carrier phase-smoothed pseudorange methods, this paper introduces a method to calculate the carrier phase change quantity $\lambda \cdot \Delta \Phi_{ij}$ (converted to distance) through the integration of the reconstructed Doppler frequency shift values. This method can be expressed as follows:

11 $$\lambda \cdot \Delta \Phi_{ij} = \lambda \left( { \Phi_{j} - \Phi_{i} } \right) = \lambda \mathop \smallint \limits_{{t_{i} }}^{{t_{j} }} f_{d} {\text{d}}t$$

In the equation, $\lambda$ represents the wavelength of the carrier signal transmitted by the satellite, $t_{i}$ and $t_{j}$ are two epochs in time, $f_{d} \left( {t_{i} } \right)$ and $f_{d} \left( {t_{j} } \right)$ represent the reconstructed Doppler frequency shift values at the two epochs, $\Phi_{i}$ and $\Phi_{j}$ denote the carrier phase observations at the two epochs, and $\lambda \cdot \Delta \Phi_{ij}$ represents the carrier phase change between the two epochs. The time difference between $t_{j} { }$ and $t_{i}$ represents the time interval between the two epochs. This approach effectively mitigates the effects of carrier phase observations including cycle slips, clock jumps, and gross errors.

Moreover, considering the carrier phase change between epochs as an indicator of the pseudorange rate, and in an ideal scenario where factors like ionospheric effects, tropospheric effects, and receiver clock bias are disregarded, the theoretical equivalence of pseudorange change and carrier phase change between the same epochs holds true. Nevertheless, in real-world scenarios, owing to diverse errors and observational noise, the carrier phase change exhibits greater precision compared to the pseudorange change. Consequently, incorporating the reconstructed Doppler frequency shift values to substitute the pseudorange change can result in a more smoothed pseudorange and enhanced pseudorange accuracy, as outlined below:

12 $$\delta \rho^{j} \left( {t_{i} ,t_{j} } \right) = \lambda \cdot \Delta \Phi_{ij} = \lambda \mathop \smallint \limits_{{t_{i} }}^{{t_{j} }} f_{d} {\text{d}}t$$

In the equation, $\delta \rho^{j} \left( {t_{i} ,t_{j} } \right)$ represents the change in pseudorange between two epochs, $t_{i}$ and $t_{j}$.

The initial epoch positioning relies on single-point pseudorange positioning, where pseudorange measurements are susceptible to significant noise. This noise is a random variable conforming to a zero-mean normal distribution, typically ranging between 5 and 10 m. Consequently, mitigating pseudorange noise becomes imperative. While traditional carrier phase-smoothed pseudorange techniques^32^ with Doppler observations show no signs of cycle slips, Doppler observations from GNSS receivers often harbor considerable noise, leading to heightened errors and diminished accuracy. In light of this, a novel approach based on reconstructed Doppler for carrier phase-smoothed pseudorange is proposed to effectively suppress pseudorange noise and enhance accuracy. The underlying principle of this method is elucidated below:

The pseudorange observations at the first epoch can be deduced by integrating the carrier phase change quantities obtained from different epochs’ reconstructed Doppler values. A smoothing window of length k is defined, which means there are k observations within the window: $\rho^{j} \left( {t_{1} } \right)$, $\rho^{j} \left( {t_{2} } \right)$, …, $\rho^{j} \left( {t_{k} } \right)$. By utilizing the integrated reconstructed Doppler frequency shift values, carrier phase change quantities $\delta \rho^{j} \left( {t_{1} ,t_{2} } \right)$, $\delta \rho^{j} \left( {t_{1} ,t_{3} } \right)$, …, $\delta \rho^{j} \left( {t_{1} ,t_{k} } \right)$ can be calculated from $t_{1}$ to $t_{k}$ and retroactively applied to the first epoch. Consequently, k pseudorange observations are available for the first epoch, namely:

13 $$\begin{aligned} \rho^{j} \left( {t_{1} } \right)_{1} = & \rho^{j} \left( {t_{1} } \right) \\ \rho^{j} \left( {t_{1} } \right)_{2} = & \rho^{j} \left( {t_{2} } \right) - \delta \rho^{j} \left( {t_{1} ,t_{2} } \right) = \rho^{j} \left( {t_{2} } \right) - \lambda \mathop \smallint \limits_{{t_{1} }}^{{t_{2} }} f_{d} {\text{d}}t \\ \cdots \cdots \\ \rho^{j} \left( {t_{1} } \right)_{k} = & \rho^{j} \left( {t_{k} } \right) - \delta \rho^{j} \left( {t_{1} ,t_{k} } \right) = \rho^{j} \left( {t_{k} } \right) - \lambda \mathop \smallint \limits_{{t_{1} }}^{{t_{k} }} f_{d} {\text{d}}t \\ \end{aligned}$$

As the sliding window encompasses *k* epochs, the pseudorange values computed for these k epochs are averaged to derive the smoothed pseudorange value for the initial epoch:

14 $$\overline{{\rho^{j} \left( {t_{1} } \right)}} = \frac{1}{k}\mathop \sum \limits_{i = 1}^{k} \rho^{j} \left( {t_{1} } \right)_{i}$$

By leveraging the integrated Doppler frequency shift values reconstructed at any specific epoch, it is possible to calculate the alteration in pseudorange concerning the initial epoch time. Following this, through the process of smoothing and adjusting the mean pseudorange value at the initial epoch time, one can obtain smoothed pseudorange values for all subsequent epochs.

15 $$\overline{{\rho^{j} \left( {t_{i} } \right)}} = \overline{{\rho^{j} \left( {t_{1} } \right)}} + \delta \rho^{j} \left( {t_{1} ,t_{i} } \right) = \overline{{\rho^{j} \left( {t_{1} } \right)}} + \lambda \mathop \smallint \limits_{{t_{1} }}^{{t_{i} }} f_{d} d$$

**Figure 2:**
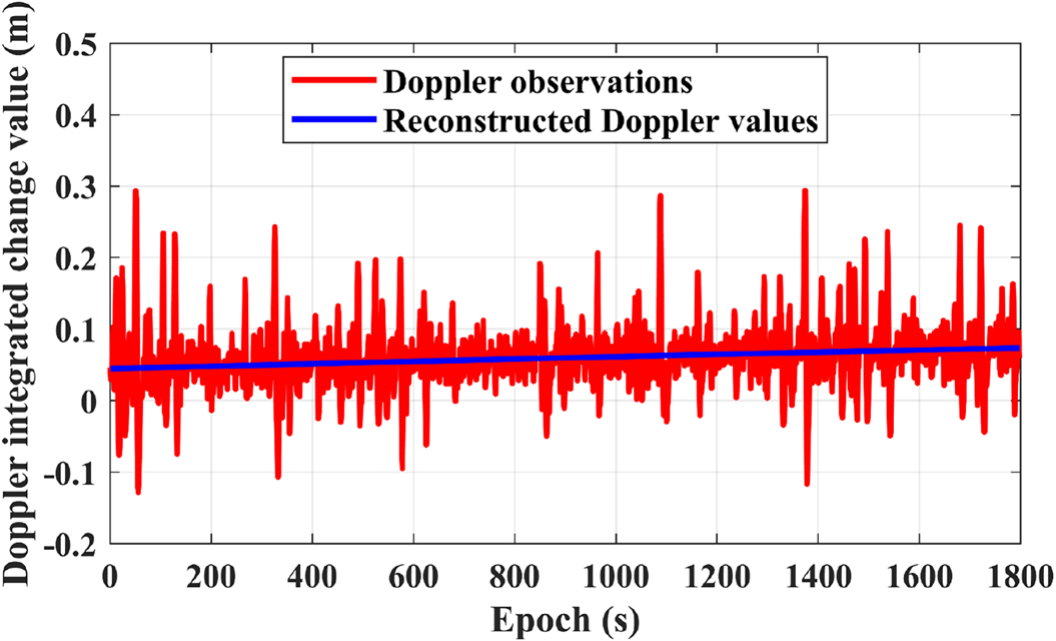
Comparison of the integrated changes between observed Doppler values and reconstructed Doppler values.

First, we integrate the Doppler values, and then obtain the integrated change by subtracting the values between consecutive epochs. This integrated change reflects the variation in noise. In Fig. 2, the comparison shows the difference between reconstructed Doppler values and Doppler observations (generated by the receiver observation file): Reconstructed Doppler values are smooth and noise-free, with a monotonically increasing curve reflecting the relative motion trajectory between the satellite and the receiver. In contrast, Doppler observation values are mixed with observational noise, displaying irregular and non-stationary fluctuations.

**Figure 3:**
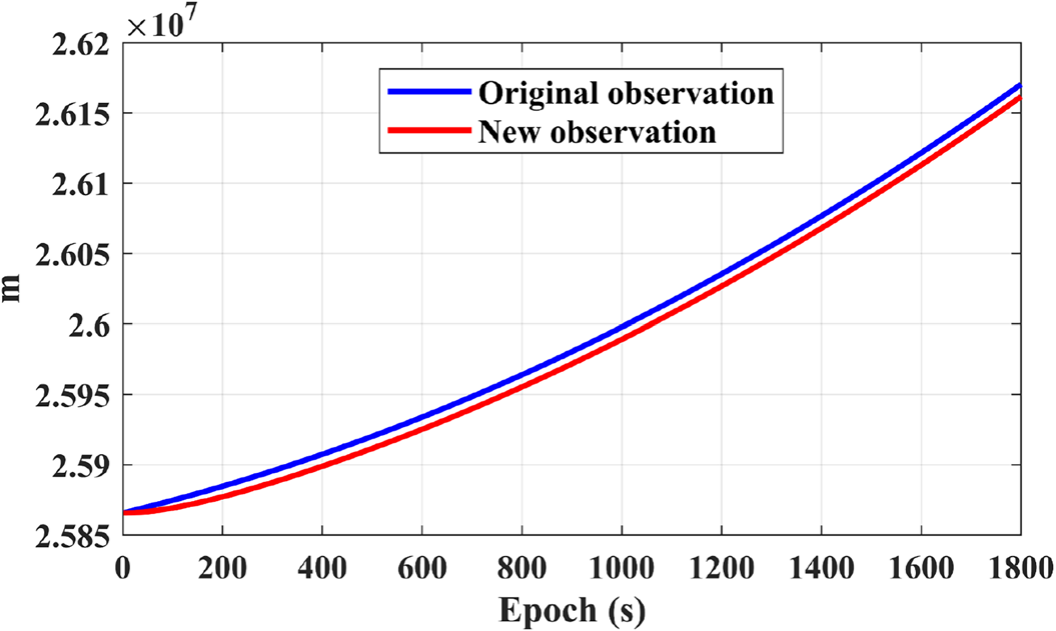
Comparison between original observations and newly generated observations.

**Figure 4:**
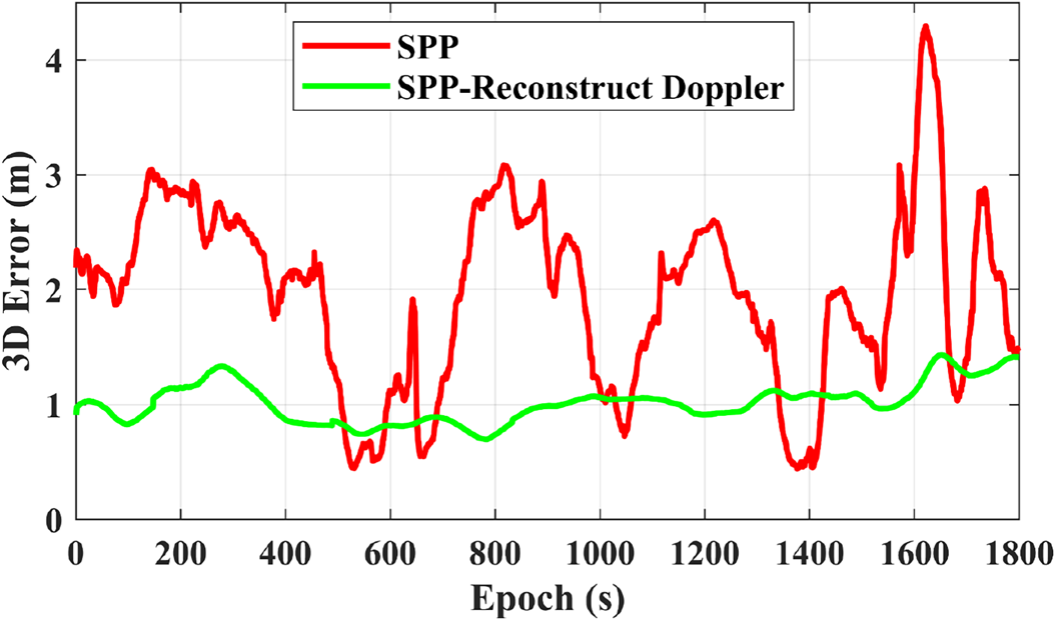
Comparison of three-dimensional positioning errors between SPP and SPP-reconstruct doppler.

Figure 3 compares the original pseudorange observations with the pseudorange observations smoothed by reconstructed Doppler. The positioning performance of these two types of observations is further validated in Fig. 4. It can be observed from Fig. 4 that the SPP-Reconstruct Doppler (the SPP with Reconstructed Doppler smoothed pseudorange) demonstrates significantly better positioning accuracy and stability compared to SPP with original pseudorange observations.

### CZS-PPP ionosphere-free combination model

The conventional PPP method employs the dual-frequency ionosphere-free model (PPP-IF), where the combined model of pseudorange and carrier phase observations is as follows:

16 $$P_{IF} = \frac{{f_{1}^{2} P_{1} - f_{2}^{2} P_{2} }}{{f_{1}^{2} - f_{2}^{2} }} = \rho + c \cdot \left( {\delta t_{k} - \delta t^{j} } \right) + G_{h} \cdot T_{h} + G_{w} \cdot T_{w} + \varepsilon_{{P_{IF} }}$$

17 $$L_{IF} = \frac{{f_{1}^{2} L_{1} - f_{2}^{2} L_{2} }}{{f_{1}^{2} - f_{2}^{2} }} = \rho + c \cdot \left( {\delta t_{k} - \delta t^{j} } \right) + G_{h} \cdot T_{h} + G_{w} \cdot T_{w} + \lambda_{IF} \cdot N_{IF} + \varepsilon_{{L_{IF} }}$$

In eqs. (16) and (17), $P_{IF}$ represents the ionosphere-free combined observations of dual-frequency pseudorange observations $P_{1}$ and $P_{2}$; $L_{IF}$ represents the ionosphere-free combined observations of dual-frequency carrier phase observations $L_{1}$ and $L_{2}$; $f_{1}$ and $f_{2}$ are the frequencies of the observations; $\delta t_{k}$ denotes the receiver clock bias; $\delta t^{j}$ represents the satellite clock bias; $c$ is the speed of light in vacuum; $G_{h}$ and $G_{w}$ are the mapping functions for dry and wet tropospheric delays, respectively; $T_{h}$ and $T_{w}$ are the dry and wet tropospheric delays; $N_{IF}$ is the integer ambiguity of the ionosphere-free combined carrier phase observations; $\lambda_{IF}$ is the wavelength of the ionosphere-free combined observations; $\varepsilon_{{P_{IF} }}$ and $\varepsilon_{{L_{IF} }}$ denote the noise errors of the pseudorange and carrier phase observations, respectively.

The dual-frequency ionosphere-free combination model^33^ for CZS-PPP is presented as follows:

18 $$P_{IF} = \frac{{f_{1}^{2} P_{1} - f_{2}^{2} P_{2} }}{{f_{1}^{2} - f_{2}^{2} }} = \rho + c \cdot \left( {\delta t_{k} - \delta t^{j} } \right) + G_{h} \cdot T_{h} + G_{w} \cdot T_{w} + \varepsilon_{{P_{IF} }}$$

19 $$\Delta L_{IF} = \frac{{f_{1}^{2} \cdot L_{1} - f_{2}^{2} \cdot L_{2} }}{{f_{1}^{2} - f_{2}^{2} }} = \Delta \rho + c \cdot \left( {\Delta \delta t_{k} - \Delta \delta t^{j} } \right) + G_{h} \cdot \Delta T_{h} + G_{w} \cdot \Delta T_{w} + \Delta \varepsilon_{{L_{IF} }}$$

In Eqs. (18) and (19), $P_{IF}$ and $\Delta L_{IF}$ represent the ionosphere-free linear combinations of pseudorange observations and carrier phase self-differencing values on BDS-3 satellites B1C and B2A, respectively. $\rho$ is the geometric distance from the receiver to the satellite, and $\Delta \rho$ represents the change in satellite-to-receiver geometric distance between the previous and current epochs. $G_{h}$ and $G_{w}$ are the tropospheric zenith direction dry and wet mapping functions, respectively. $\Delta T_{h}$ is the change in tropospheric zenith direction dry delay component between the previous and current epochs, and $\Delta T_{w}$ is the change in tropospheric zenith direction wet delay component between the previous and current epochs. $\Delta T_{w}$ is treated as an estimated parameter along with position parameters. $\varepsilon_{{P_{IF} }}$ represents the pseudorange observation noise for the ionosphere-free combination, while $\Delta \varepsilon_{{L_{IF} }}$ represents the phase observation noise for the ionosphere-free combination between the previous and current epochs. All the above units are in meters. *c* denotes the speed of light. $\Delta \delta t_{k}$ is the change in receiver clock bias between the previous and current epochs, and ${\Delta }\delta t^{j}$ is the change in satellite clock bias between the previous and current epochs, both in seconds. Other error terms in the observation equation (such as antenna phase center corrections^34^, phase wind-up effects^35^, tidal loading deformations^36^, relativistic effects^37^, and Earth rotation^38^) are corrected using respective models. The dual-frequency ionosphere-free combination model offers the advantages of eliminating first-order ionospheric effects with fewer estimated parameters, resulting in stable positioning performance, a simple model, and ease of operation.

In our research, we utilized $L_{1}$ and $L_{2}$ dual-frequency observations from the BDS-3 and GPS dual-systems^39^ to compute the intermediate-frequency combinations. The ionosphere-free combination equation, established based on pseudorange observations and carrier phase self-differencing values, can be expressed as:

20 $$P_{IF}^{g} = \rho^{g} + c \cdot \delta t_{k} + G_{w} \cdot T_{w}^{g} + \varepsilon_{{P_{IF} }}^{^{\prime}g}$$

21 $$\Delta L_{IF}^{g} = \Delta \rho^{g} + c \cdot \Delta \delta t_{k} + G_{w} \cdot \Delta T_{w}^{g} + \Delta \varepsilon_{{L_{IF} }}^{^{\prime}g}$$

22 $$P_{IF}^{b} = \rho^{b} + c \cdot \delta t_{k} + G_{w} \cdot T_{w}^{b} + c \cdot \delta t_{sys}^{b,g} + \varepsilon_{{P_{IF} }}^{^{\prime}b}$$

23 $$\Delta L_{IF}^{b} = \Delta \rho^{b} + c \cdot \Delta \delta t_{k} + G_{w} \cdot \Delta T_{w}^{b} + c \cdot \Delta \delta t_{sys}^{b,g} + \Delta \varepsilon_{{L_{IF} }}^{^{\prime}b}$$

In the equations, ${\Delta }$ represents the differencing operator between the previous and current epochs. The superscripts $g$ and $b$ denote GPS and BDS-3 satellites, respectively. $\delta t_{sys}^{b,g}$ represents the system time difference parameter between GPS and BDS-3, measured in seconds. In the dual-constellation PPP model combining GPS and BDS-3, in this paper, GPS time is used as the reference and BDS-3 time is converted to GPS time. In addition, we include the parameter $\Delta \delta t_{sys}^{b,g}$ in the set of parameters to be determined for estimating the system clock difference between GPS and BDS-3. The dual-frequency ionosphere-free combination model consists of five parameters: position changes ($\Delta x,\Delta y,\Delta z$), receiver clock bias $\Delta \delta t_{k}$, tropospheric zenith wet component $\Delta T_{w}$, and system time difference parameter $\Delta \delta t_{sys}^{b,g}$.

24 $${\text{d}}X = \left[ {\Delta x,\Delta y,\Delta z,\Delta \delta t_{k} ,\Delta T_{w} ,{ }\Delta \delta t_{sys}^{b,g} } \right]$$

In this paper, a conventional random model is used, and the noise ($\sigma$) in the observation equation is weighted according to the elevation angle.

25 $$\left\{ {\begin{array}{*{20}l} {\sigma \left( {ele} \right) = \sigma_{0} } \hfill & {ele > 30^{^\circ } } \hfill \\ {\sigma \left( {ele} \right) = \frac{{\sigma_{0} }}{{2\sin \left( {ele} \right)}} } \hfill & {else } \hfill \\ \end{array} } \right.$$

In the Eq. (25), $ele$ is the elevation angle. $\sigma_{0}$ is commonly set to 1 m for pseudorange observations and 1 cm for phase observations. The random model is as follows:

26 $$w = R^{ - 1} = \left[ {\begin{array}{*{20}c} {\frac{1}{{\sigma_{1}^{2} }}} & {} & {} \\ {} & \ddots & {} \\ {} & {} & {\frac{1}{{\sigma_{n}^{2} }}} \\ \end{array} } \right]$$

In the Eq. (26), $w$ signifies the weight matrix of the observations, while $R$ denotes the covariance matrix associated with the observations.

Due to the different observation accuracies of GPS and BDS, the corresponding observation weights are also different. In this paper the observation weights of GPS and BDS are set to 1:2.

## Algorithm validation and performance analysis

The method in this paper is applicable to static mode, in order to verify the effectiveness of the above CZS-PPP algorithm for static data processing, experiments will be carried out utilizing a Beidou Xingtong receiver at the Intelligent Navigation and Remote Sensing Research Center of Xiangtan University. All receivers employed in these experiments will utilize both the GPS and BDS-3 satellite navigation systems for precise positioning.

**Figure 5:**
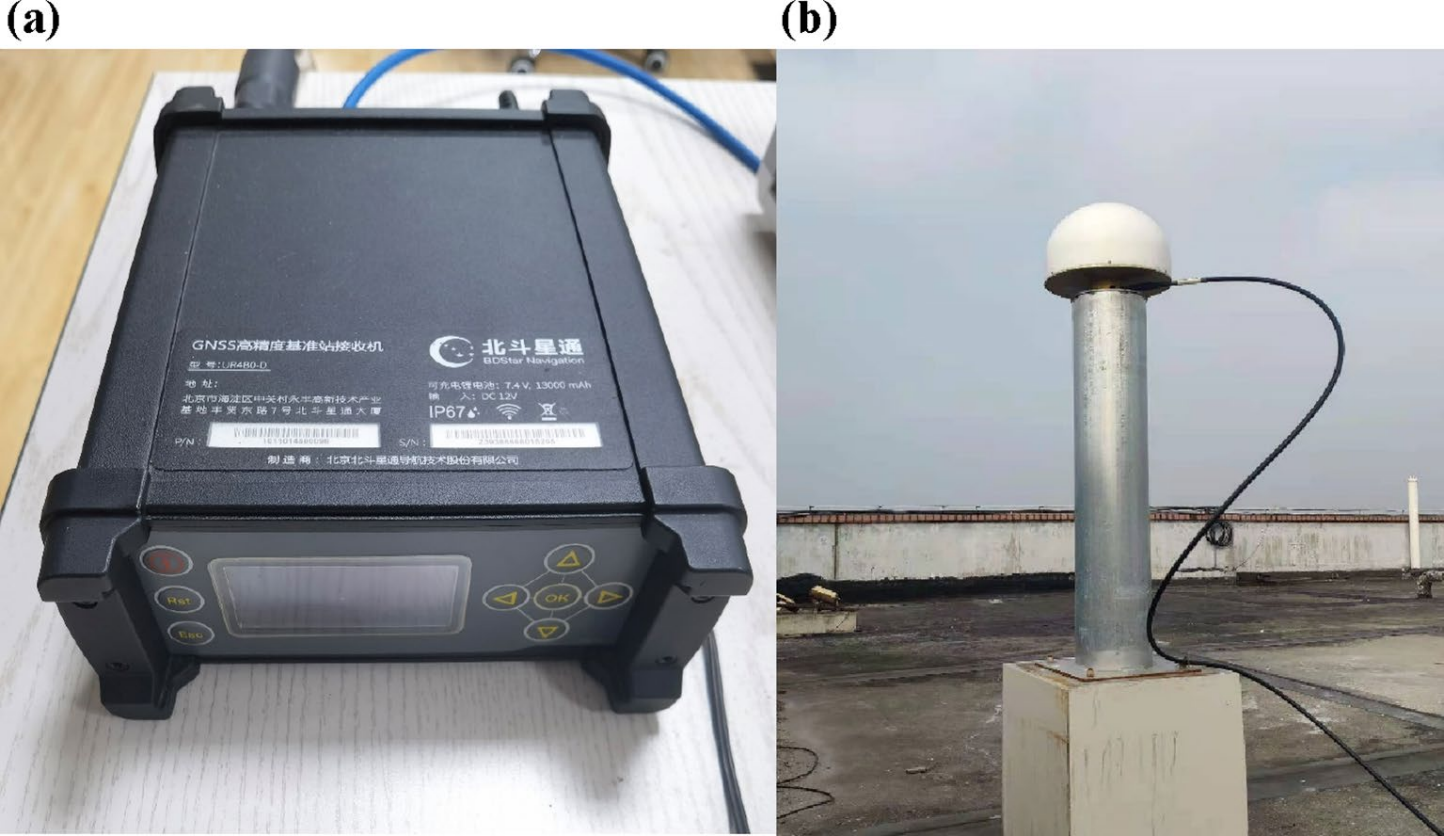
(**a**) Beidou Xingtong Receiver and (**b**) Beidou Xingtong Antenna.

As depicted in Fig. 5, the algorithmic experiments employed a dual-frequency GNSS receiver (BeiDou Xingtong) positioned on the rooftop of the Information Building at Xiangtan University to capture satellite signals. This setup significantly reduced multipath effects, which could be considered negligible. The Earth Centered, Earth Fixed (ECEF) coordinates of its antenna were accurately measured using standard precision instruments, achieving an accuracy better than 3 mm. Original observation data spanning days 017–023 of the year 2023, totaling 7 days, was acquired. The data was sampled at 1-s intervals, encompassing simultaneous reception of BDS-3 and GPS satellite data. Raw observation data was collected daily from 9:00 to 10:00, over a one-hour period. The convergence criteria in this paper are as follows: achieving convergence thresholds simultaneously in the East (E), North (N), and Up (U) directions for PPP, and ensuring stability within the thresholds for 120 consecutive epochs (with a sampling interval of 60 min). The convergence threshold is set at 10 cm, and the convergence time is measured from the initial epoch to reaching the convergence threshold. The experiment utilized dual-frequency raw observation data from BDS-3 and GPS satellite systems to validate the superiority of CZS-PPP.

### Experiment 1

In conditions where only a single system was considered, distinct experiments were conducted to validate and compare the individual BDS-3 system^40,41^ and the standalone GPS system. Precise satellite coordinates and accurate satellite clock biases were sourced from products provided by the International GNSS Service (IGS) center. CZS-PPP utilized 5-min precise satellite coordinates and 30-s precise satellite clock bias products from the German Research Centre for Geosciences (GFZ). Raw observations in dual frequency, encompassing BDS-3 B1C, B2A^42,43^, and GPS L1C, L2W, were collected over seven consecutive days, from the 17th to the 23rd day of the year 2023, as acquired by the receiver.

**Figure 6:**
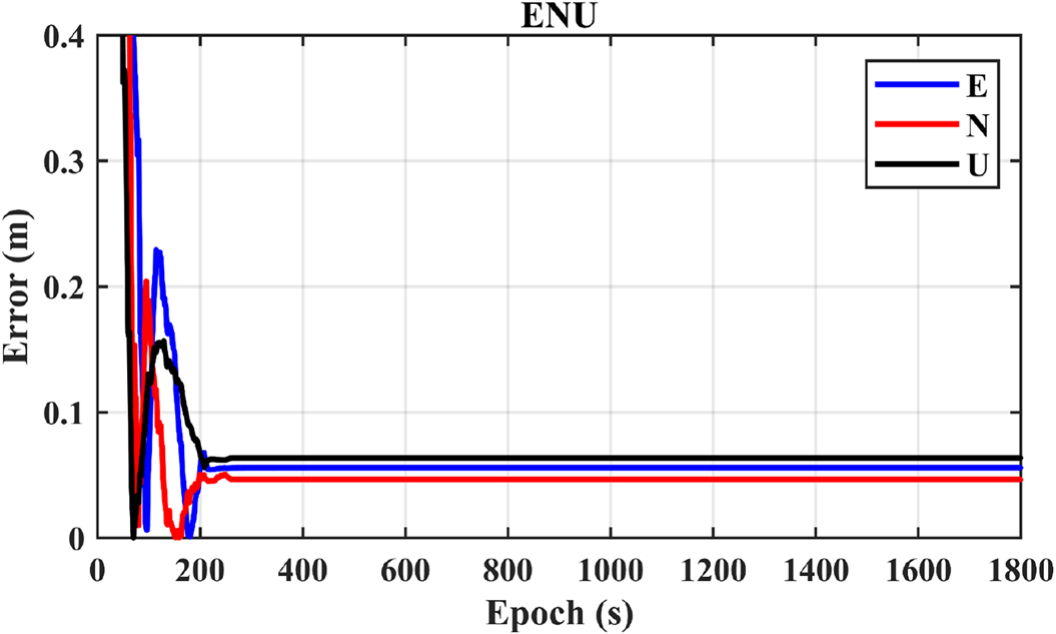
Positioning results for a single BDS-3 system.

**Figure 7:**
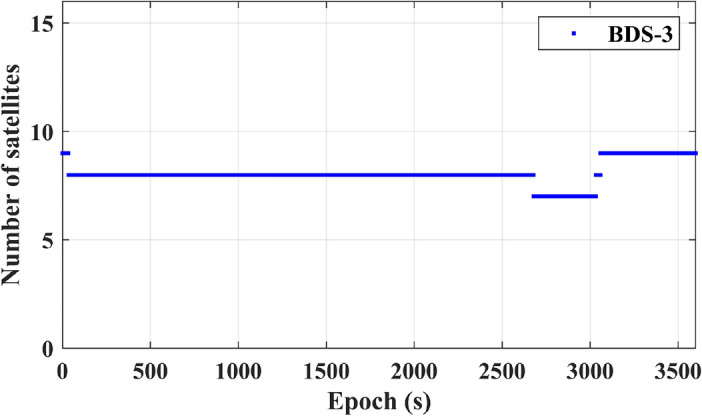
BDS-3 satellite observations.

Figure 6 showcases the positioning results for a single BDS-3 system. In the diagram, (E, N, U) represent a specific station coordinate system, known as the East-North-Up (ENU) coordinate system. The ENU coordinate system captures the positional changes of the receiver in the East (E), North (N), and Up (U) directions. The error curves in the figure depict the absolute differences between the coordinates obtained through CZS-PPP calculations and the receiver’s standard coordinates in the ENU coordinate systems. Over time, convergence is achieved. At 217 s, the convergence reaches 0.07 m in the E direction, 0.06 m in the N direction, and 0.08 m in the U direction. Figure 7 illustrates the number of BDS-3 satellites observed within an hour. During the specified time interval, approximately 8 satellites are observed, meeting the minimum requirement of at least 4 satellites for positioning.

**Figure 8:**
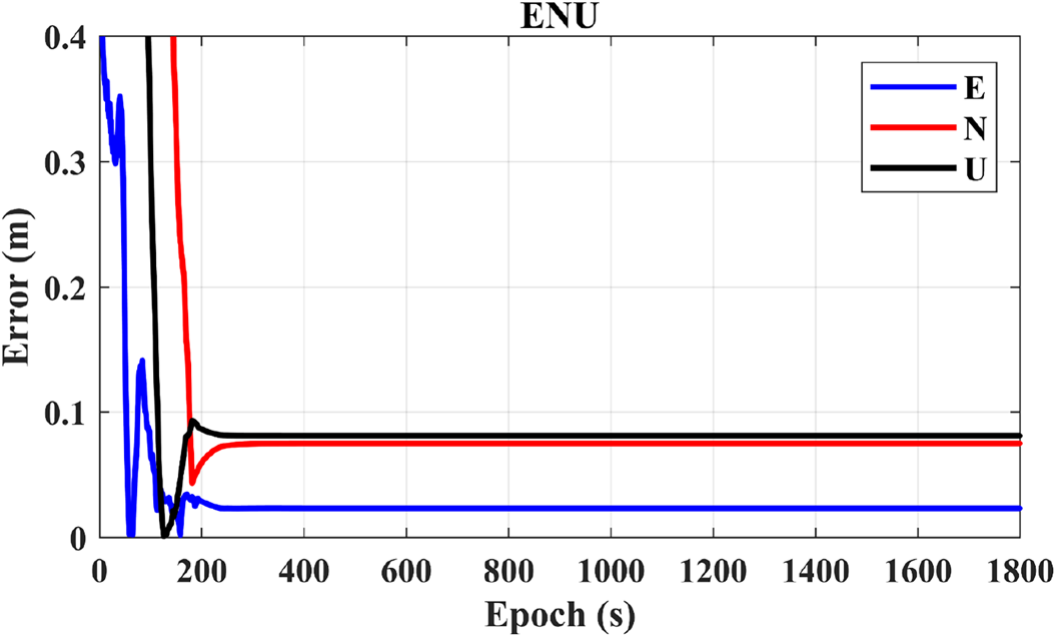
Presents the positioning results for a single GPS system.

**Figure 9:**
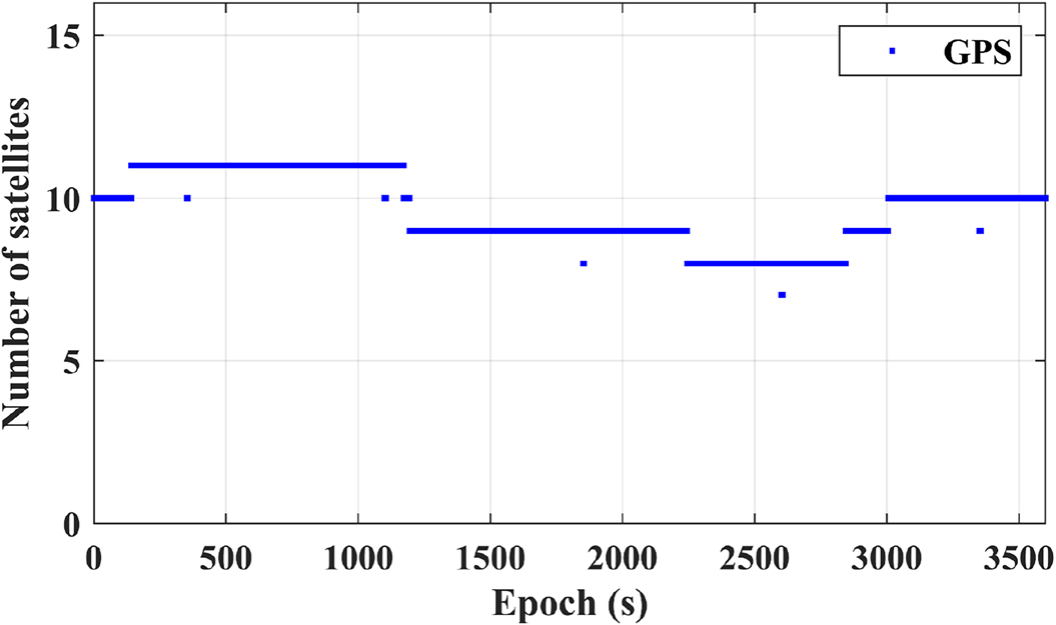
Displays the number of observed GPS satellites.

Figure 8 depicts the positioning results for a single GPS system. Over time, convergence is attained, reaching 0.03 m in the E direction, 0.08 m in the N direction, and 0.09 m in the U direction at 201 s. Following convergence, the positioning accuracy remains consistently stable. In contrast, Fig. 9 illustrates that the number of GPS satellites is approximately equivalent to the number of BDS-3 satellites.

Table 1 presents information on the convergence time and three-dimensional RMS positioning accuracy in the ENU coordinates for both the individual BDS-3 satellite system and the standalone GPS satellite system. Based on continuous observations spanning 7 days, it is evident that the average convergence time for the single BDS-3 system is 220 s, accompanied by an average ENU three-dimensional RMS positioning accuracy of 0.08 m. In comparison, the single GPS system demonstrates an average convergence time of 214 s, with an average ENU three-dimensional RMS positioning accuracy of 0.076 m. The two satellite systems display comparable average convergence times and average positioning accuracy.

**Table 1:** Comparison between BDS-3 and GPS systems.

Statistic (Annual date)	BDS-3		GPS	
	Convergence(s) RMS(m)	ENU 3D	Convergence(s) RMS(m)	ENU 3D
017	213	0.08	201	0.07
018	229	0.09	200	0.10
019	205	0.07	219	0.06
020	233	0.10	213	0.10
021	223	0.10	198	0.05
022	225	0.05	237	0.08
023	217	0.07	221	0.07
Mean	220	0.08	214	0.076

### Experiment 2

In the context of the BDS-3/GPS dual-satellite system^44,45^, the positioning results of CZS-PPP are compared with those of the widely employed PPP method. The conventional PPP method employs the PPP-IF model^46,47^. Both methodologies utilize identical observational data and precise ephemeris. A comparative analysis is conducted using data continuously collected for 7 days, drawn from the dual-frequency raw data received by the receiver over the consecutive 7 days, spanning from day 17 to day 23 in 2023.

**Figure 10:**
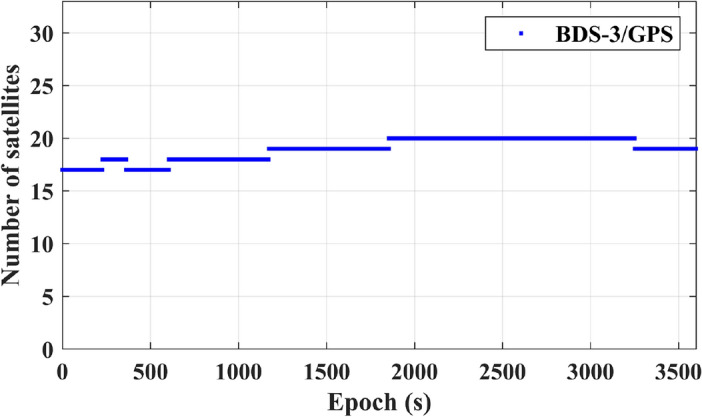
Number of BDS-3/GPS dual-system satellite observations.

In Fig. 10, in the context of a dual-satellite navigation system, a notable increase in the count of visible satellites is depicted, leading to an enhanced satellite spatial geometry.

**Figure 11:**
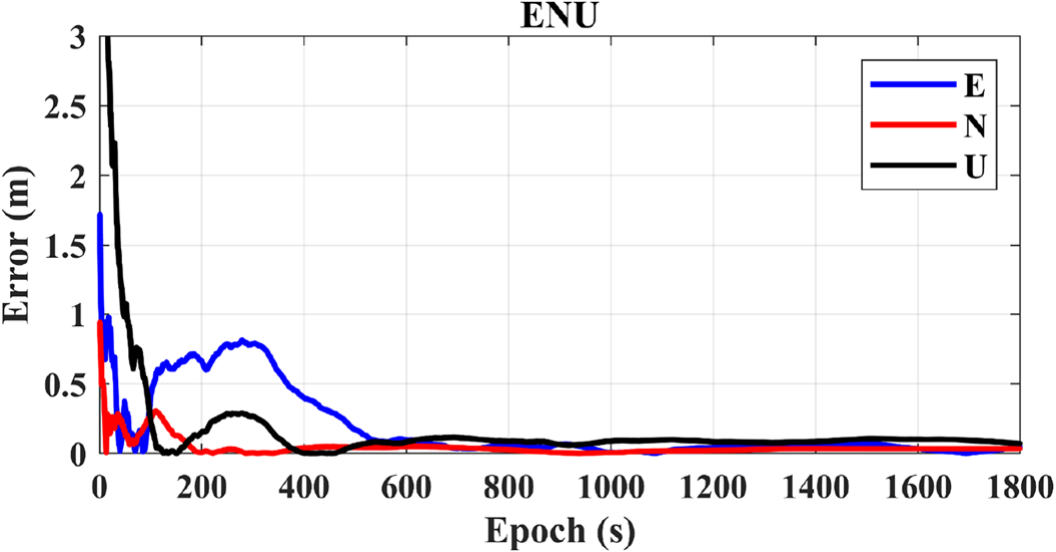
PPP-IF positioning results.

In Fig. 11, it is evident that the convergence time for PPP-IF is notably extended, requiring approximately 700 s to converge, and achieving a three-dimensional average positioning accuracy of 0.15 m.

**Figure 12:**
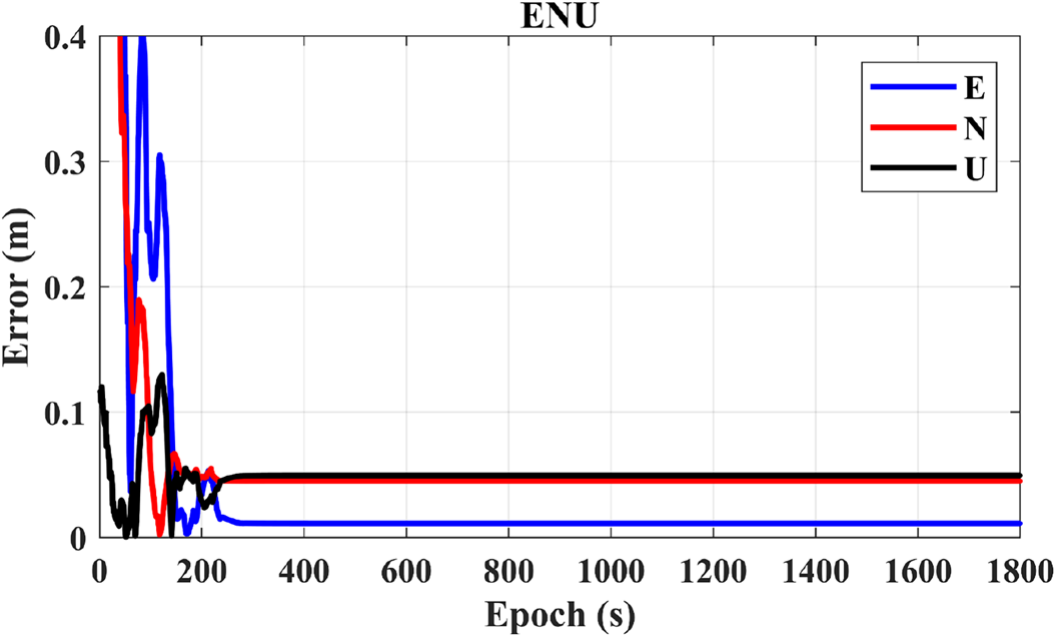
Positioning results of CZS-PPP.

The outcomes illustrated in Fig. 12 clearly demonstrate the superior performance of CZS-PPP over PPP-IF. CZS-PPP achieves convergence in 152 s and sustains a three-dimensional average positioning accuracy of 0.06 m. This highlights the superior performance of CZS-PPP in terms of both convergence time and positioning accuracy when compared to PPP-IF.

**Figure 13:**
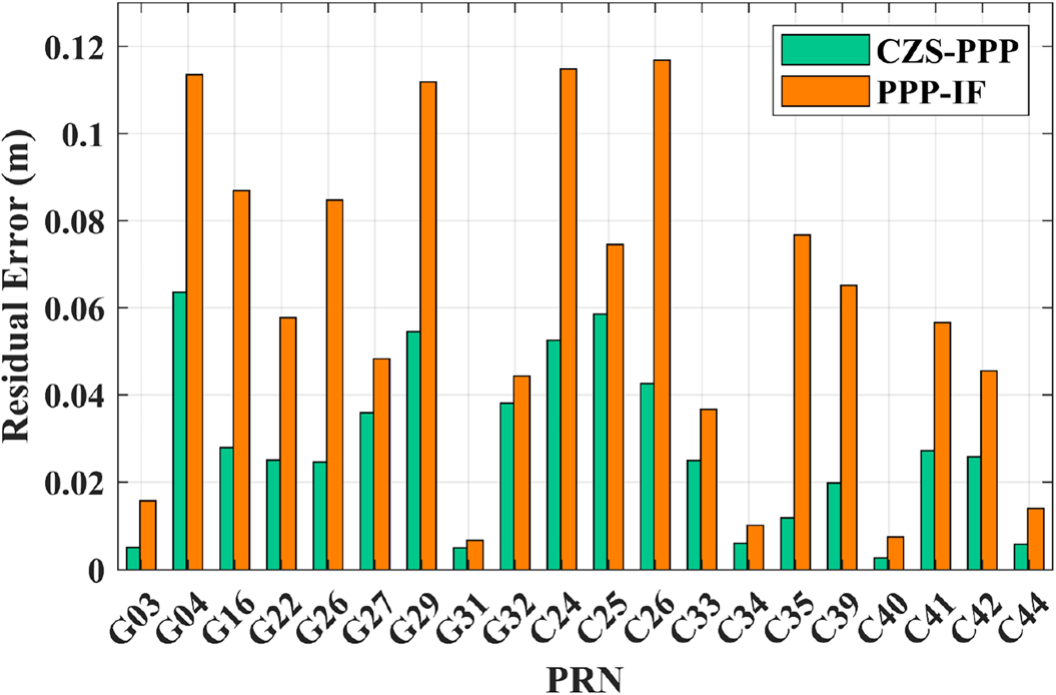
Comparison of system residual errors between CZS-PPP and PPP-IF.

In Fig. 13, this paper presents a statistical comparison of the system residual errors between CZS-PPP and the traditional PPP-IF method under the dual-constellation conditions of GPS and BDS-3. The horizontal axis represents satellite numbers, and the vertical axis represents system residual errors. It can be observed that the system residual errors of the proposed method are significantly smaller than those of the traditional PPP-IF method.

Table 2 presents data spanning seven consecutive days. The average convergence time for PPP-IF is 709 s, accompanied by an average three-dimensional RMS positioning accuracy of 0.132 m. In contrast, CZS-PPP attains an average convergence time of 156 s, with an average three-dimensional RMS positioning accuracy of 0.068 m. This underscores the superior performance of CZS-PPP.

**Table 2:** Comparison between CZS-PPP and PPP-IF.

Statistic (Annual date)	PPP-IF		CZS-PPP	
	Convergence(s) RMS(m)	ENU 3D	Convergence(s) RMS(m)	ENU 3D
017	688	0.11	147	0.05
018	732	0.16	168	0.09
019	714	0.10	159	0.05
020	722	0.12	162	0.06
021	700	0.15	152	0.07
022	681	0.12	143	0.06
023	729	0.17	165	0.10
Mean	709	0.132	156	0.068

## Conclusions

This study delves into the critical factors impeding the rapid convergence of PPP, such as integer ambiguity and residual errors within the system. Notably, it addresses these challenges without relying on external sources like ground augmentation networks or enhancements from low Earth orbit satellite navigation systems, resulting in a significant reduction in PPP convergence time. The proposed method, termed Carrier Phase Zero-Baseline Self-Differencing PPP (CZS-PPP), initially employs principles of satellite precise orbits and Doppler generation to reconstruct error-free Doppler measurements. It then utilizes Doppler integration equations to smooth pseudorange, minimizing cumulative residual errors and enhancing pseudorange accuracy for precise initial epoch coordinates. Subsequently, through self-differencing between consecutive epochs, system residual errors and integer ambiguities are further mitigated, leading to a substantial reduction in PPP convergence time. The single-system case achieves convergence in less than 4 min, while the dual-system case converges in under 3 min. The accuracy of these findings is effectively validated using actual measurements from BDS-3, GPS, and BDS-3/GPS. This research contributes crucial theoretical and technical support for real-world applications of BDS-3/GNSS real-time PPP.

## Data Availability

The data related to or connected with the work are all encompassed in the manuscript and its supporting information. The datasets generated and/or analysed during the current study are not publicly available due the confidentiality of this project but are available from the corresponding author on reasonable request.

## References

[1] Elsobeiey M, El-Rabbany A (2014). Efficient between-satellite single-difference precise point positioning model. J. Surv. Eng..

[2] Afifi A, El-Rabbany A (2016). Improved between-satellite single-difference precise point positioning model using triple GNSS constellations: GPS, Galileo, and BeiDou. Positioning.

[3] Zhao Q, Pan S, Gao W (2022). Multi-GNSS fast precise point positioning with multi-frequency uncombined model and cascading ambiguity resolution. Math. Probl. Eng..

[4] Zangenehnejad, F., Gao, Y. Application of UofC model based multi-GNSS PPP to smartphones GNSS positioning. In: proceedings of the 34th international technical meeting of the satellite division of the institute of navigation (ION GNSS+ 2021). 2021: 2986–3003.

[5] Odijk D (2003). Ionosphere-free phase combinations for modernized GPS. J. Surv. Eng..

[6] Srinivas, V.S., Yedukondalu, K. Code-phase based combined GPS-Galileo positioning using Ionosphere-free linear combination//2019 URSI Asia-Pacific Radio Science Conference (AP-RASC). IEEE, 2019: 1–4.

[7] Schlüter S, Hoque MM (2020). An SBAS integrity model to overbound residuals of higher-order ionospheric effects in the Ionosphere-free linear combination. Remote Sens..

[8] Banville S (2016). GLONASS ionosphere-free ambiguity resolution for precise point positioning. J. Geod..

[9] Zhang W, Lou Y, Song W (2022). Initial assessment of BDS-3 precise point positioning service on GEO B2b signal. Adv. Space Res.: Off. J. Comm. Space Res..

[10] Nie Z, Xu X, Wang Z (2021). Initial assessment of BDS PPP-B2b service: Precision of orbit and clock corrections, and PPP performance. Remote Sens..

[11] Tang C, Hu X, Chen J (2022). Orbit determination, clock estimation and performance evaluation of BDS-3 PPP-B2b service. J. Geod..

[12] Xu Y, Yang Y, Li J (2021). Performance evaluation of BDS-3 PPP-B2b precise point positioning service. GPS Solut..

[13] Ren Z, Gong H, Peng J (2021). Performance assessment of real-time precise point positioning using BDS PPP-B2b service signal. Adv. Space Res..

[14] Guo J, Zhang Q, Li G (2021). Assessment of multi-frequency PPP ambiguity resolution using Galileo and BeiDou-3 signals. Remote Sens.ng.

[15] Duong V, Harima K, Choy S (2020). Assessing the performance of multi-frequency GPS, Galileo and BeiDou PPP ambiguity resolution. J. Spat. Sci..

[16] Laurichesse D, Banville S (2018). Instantaneous centimeter-level multi-frequency precise point positioning. GPS World Innov. Column.

[17] Li J, Yang Y, He H (2017). An analytical study on the carrier-phase linear combinations for triple-frequency GNSS. J. Geod..

[18] Basile F, Moore T, Hill C (2020). GPS and galileo triple-carrier ionosphere-free combinations for improved convergence in precise point positioning. J. Navig..

[19] Li P, Zhang X, Ge M (2018). Three-frequency BDS precise point positioning ambiguity resolution based on raw observables. J. Geod..

[20] Bu J, Yu K, Qian N (2020). Performance assessment of positioning based on multi-frequency multi-GNSS observations: Signal quality, PPP and baseline solution. IEEE Access.

[21] Xu W, Shen W, Cai C (2022). Comparison and evaluation of carrier phase PPP and single difference time transfer with multi-GNSS ambiguity resolution. GPS Solut..

[22] Jianghui G, Hua C, General G, Guangcai L, Na W (2020). Three multi-frequency and multi-system GNSS single-point high-precision positioning methods and performance analysis for complex urban environments. J. Surv. Mapp..

[23] Li X, Li X, Yuan Y (2018). Multi-GNSS phase delay estimation and PPP ambiguity resolution: GPS, BDS, GLONASS, Galileo. J. Geod..

[24] Duong V, Harima K, Choy S (2019). An optimal linear combination model to accelerate PPP convergence using multi-frequency multi-GNSS measurements. GPS Solut..

[25] Khodabandeh A, Teunissen PJG (2015). An analytical study of PPP-RTK corrections: Precision, correlation and user-impact. J. Geod..

[26] Xiaohong Z, Jiahuan H, Xiaodong R (2020). Comparison of PPP/PPP-RTK new progress and Beidou/GNSS PPP positioning performance. J. Surv. Mapp..

[27] Li X, Li X, Huang J (2021). Improving PPP–RTK in urban environment by tightly coupled integration of GNSS and INS[J]. J. Geod..

[28] Fan C, Yao Z, Yun S (2021). Ground-based PPP-RTK for pseudolite systems. J. Geod..

[29] Ge H, Li B, Ge M (2018). Initial assessment of precise point positioning with LEO enhanced global navigation satellite systems (LeGNSS). Remote Sens..

[30] Li X, Ma F, Li X (2019). LEO constellation-augmented multi-GNSS for rapid PPP convergence. J. Geod..

[31] Saastamoinen J (1972). Atmospheric correction for the troposphere and stratosphere in radio ranging satellites. Use Artif. Satell. Geod..

[32] Zengkai, S., Xurong, D., Yanfeng, H., *et al*. Development of Doppler-assisted improvement of pseudorange/carrier phase accuracy. In: proceedings of the 12th annual China satellite navigation conference - S07 satellite navigation enhancement technology, 2021.

[33] Feiyang, W. & Li, X. Estimation and new characterization of phase fractional deviation of BeiDou-3 based on different PPP models[J]. *Global Positioning System.* **48**(01), 14–23 (2023)

[34] Zhou R, Hu Z, Zhao Q (2022). Consistency analysis of the GNSS antenna phase center correction models. Remote Sens..

[35] Wu JT, Wu SC, Hajj GA (1991). Effects of antenna orientation on GPS carrier phase. Astrodynamics.

[36] Zhao H, Zhang Q, Tu R (2018). Determination of ocean tide loading displacement by GPS PPP with priori information constraint of NAO99b global ocean tide model. Mar. Geod..

[37] Hećimović Ž (2013). Relativistic effects on satellite navigation. Tech. Gazette.

[38] Hu, W. & Farrell, J. A. Technical Note: Derivation of Earth-Rotation Correction (Sagnac) and Analysis of the Effect of Receiver Clock Bias. (2019).

[39] Yaqiong, H. Research on key issues of precision single-point localization with BDS/GPS dual system [D]. University of Chinese Academy of Sciences (Institute of Precision Measurement Science and Technology Innovation, Chinese Academy of Sciences). 10.27602/d.cnki.gkwws.2021.000073 (2021).

[40] Zhang K, Guo G (2019). Analysis of BDS-3 satellite performance enhancement for BDS global positioning. Global Position. Syst..

[41] Zhu Y, Zhang Q, Mao Y (2022). Comprehensive performance review of BDS-3 after one-year official operation. Adv. Space Res..

[42] Ye F, Yuan Y, Yang Z (2022). Validation and evaluation on B1IB3I-based and B1CB2a-based BDS-3 precise orbits from iGMAS. Adv. Space Res..

[43] Zhang Q, Zhu Y, Chen Z (2021). An in-depth assessment of the new BDS-3 B1C and B2a signals. Remote Sens..

[44] Wang M, Shan T, Zhang W (2021). Analysis of BDS/GPS signals’ characteristics and navigation accuracy for a geostationary satellite. Remote Sens..

[45] Yangfei, H., Junping, C., Yize, Z. (2022). PPP floating-point solution and fixed solution positioning accuracy analysis of BDS-3 new frequency (B1C+B2a) fused with GPS. In: proceedings of the 13th annual China satellite navigation conference. 1–6.

[46] Zhang R, Gao C, Wang Z (2022). Ambiguity resolution for long baseline in a network with BDS-3 quad-frequency ionosphere-weighted model. Remote Sens..

[47] Jie L, Rongzhi Z, Guang Z (2020). Clock difference estimation of two ionosphere-free combined orbits from BeiDou tri-frequency data and its application. J. Surv. Mapp..

