# Author Correction: A static precise single-point positioning method based on carrier phase zero-baseline self-differencing

**DOI:** 10.1038/s41598-024-66587-9

**Published:** 2024-07-10

**Authors:** Kaihui Lv, Chenglin Cai, Yihao Cai, Wenhui Guan, Zexian Li, Mingjie Wu, Lingfeng Cheng

**Affiliations:** 1 School of Mathematics and Computational Science, Xiangtan University, Xiangtan, 411105 Hunan China; 2 School of Automation and Electronic Information, Xiangtan University, Xiangtan, 411105 Hunan China; 3 School of Materials Science and Engineering, Xiangtan University, Xiangtan, 411105 Hunan China; 4 Shanghai Astronomical Observatory, Chinese Academy of Sciences, Shanghai, 200030 China; 5 School of Physical Science and Technology, ShanghaiTech University, Shanghai, 200030 China

Correction to: *Scientific Reports* 10.1038/s41598-024-63570-2, published online 1 June 2024

In the original version of this Article, Chenglin Cai was omitted as a corresponding author. Correspondence and requests for materials should also be addressed to chengcailin@126.com.

It also contained an error in the order of the author names, which was incorrectly given as Chenglin Cai, Kaihui Lv, Yihao Cai, Wenhui Guan, Zexian Li, Mingjie Wu and Lingfeng Cheng.

The original Article has been corrected.

